# Model-Based Reasoning of Clinical Diagnosis in Integrative Medicine: Real-World Methodological Study of Electronic Medical Records and Natural Language Processing Methods

**DOI:** 10.2196/23082

**Published:** 2020-12-21

**Authors:** Wenye Geng, Xuanfeng Qin, Tao Yang, Zhilei Cong, Zhuo Wang, Qing Kong, Zihui Tang, Lin Jiang

**Affiliations:** 1 Department of Integrative Medicine Fudan University Huashan Hospital Shanghai China; 2 Department of Neurosurgery Fudan University Huashan Hospital Shanghai China; 3 Emergency Department Huashan Hospital of Fudan University Shanghai China; 4 Shanghai Sunjian Informatics Technology Company Limited Shanghai China; 5 Healthcare Center Fudan University Huashan Hospital Shanghai China

**Keywords:** model-based reasoning, integrative medicine, electronic medical records, natural language processing

## Abstract

**Background:**

Integrative medicine is a form of medicine that combines practices and treatments from alternative medicine with conventional medicine. The diagnosis in integrative medicine involves the clinical diagnosis based on modern medicine and syndrome pattern diagnosis. Electronic medical records (EMRs) are the systematized collection of patients health information stored in a digital format that can be shared across different health care settings. Although syndrome and sign information or relative information can be extracted from the EMR and content texts can be mapped to computability vectors using natural language processing techniques, application of artificial intelligence techniques to support physicians in medical practices remains a major challenge.

**Objective:**

The purpose of this study was to investigate model-based reasoning (MBR) algorithms for the clinical diagnosis in integrative medicine based on EMRs and natural language processing. We also estimated the associations among the factors of sample size, number of syndrome pattern type, and diagnosis in modern medicine using the MBR algorithms.

**Methods:**

A total of 14,075 medical records of clinical cases were extracted from the EMRs as the development data set, and an external test data set consisting of 1000 medical records of clinical cases was extracted from independent EMRs. MBR methods based on word embedding, machine learning, and deep learning algorithms were developed for the automatic diagnosis of syndrome pattern in integrative medicine. MBR algorithms combining rule-based reasoning (RBR) were also developed. A standard evaluation metrics consisting of accuracy, precision, recall, and F1 score was used for the performance estimation of the methods. The association analyses were conducted on the sample size, number of syndrome pattern type, and diagnosis of lung diseases with the best algorithms.

**Results:**

The Word2Vec convolutional neural network (CNN) MBR algorithms showed high performance (accuracy of 0.9586 in the test data set) in the syndrome pattern diagnosis of lung diseases. The Word2Vec CNN MBR combined with RBR also showed high performance (accuracy of 0.9229 in the test data set). The diagnosis of lung diseases could enhance the performance of the Word2Vec CNN MBR algorithms. Each group sample size and syndrome pattern type affected the performance of these algorithms.

**Conclusions:**

The MBR methods based on Word2Vec and CNN showed high performance in the syndrome pattern diagnosis of lung diseases in integrative medicine. The parameters of each group’s sample size, syndrome pattern type, and diagnosis of lung diseases were associated with the performance of the methods.

**Trial Registration:**

ClinicalTrials.gov NCT03274908; https://clinicaltrials.gov/ct2/show/NCT03274908

## Introduction

Integrative medicine is a form of medicine that combines practices and treatments from alternative medicine with conventional medicine [1-3]. In China, integrative medicine combines traditional Chinese medicine (TCM) and modern medicine for clinical practice [1-3]. The diagnosis in integrative medicine comprises the clinical diagnosis based on modern medicine and syndrome pattern diagnosis [4]. Syndrome pattern based on TCM theory is an outcome of the analysis of TCM information by the TCM practitioner, and TCM treatments rely on this concept [4]. A syndrome pattern can be defined as a categorized pattern of symptoms and signs in a patient at a specific stage during the course of a disease. Syndrome elements are the smaller units of syndrome classification and the basic elements of a syndrome pattern [5]. The correct combination of syndrome elements can infer an appropriate syndrome pattern. Syndrome elements are also derived from the syndrome and signs from the patient [5,6]. Generally, practitioners of integrative medicine making diagnosis decisions need to combine syndrome pattern diagnosis and the diagnosis in modern medicine [5,6]. As TCM treatments rely on syndrome pattern diagnosis, the treatment combined with the therapies of TCM and modern medicine is expected to be more efficient for patients. Therefore, syndrome pattern for the diagnosis in integrative medicine is an essential part of diagnosis.

Electronic medical records (EMRs) are the systematized collection of patients’ and the population’s electronically stored health information in a digital format that can be shared across different health care settings [7,8]. In China, EMRs are a collection of diagnoses of syndrome patterns and model medicine as well as syndromes and signs with the TCM format [7,8]. Natural language processing (NLP) is a field of artificial intelligence and computational linguistics concerned with the interactions between computers and human natural languages [9,10]. Currently, NLP techniques combining EMRs have been comprehensively applied to medical data mining and medical decision support system [9,10]. Word embedding, as one of the techniques in NLP, attempted to map a word using a dictionary to a vector of real numbers in a low-dimensional space [11,12]. It is important in EMR data mining or artificial intelligence application in medicine for medical texts to be transferred to vectors because computers can handle or understand medical texts through computability vectors.

Applying artificial intelligence techniques to support physicians in medical practices is a major challenge. The processing of uncertainty information mainly contributes to the challenge. Syndrome and sign information is under the classic uncertainty information. The artificial neural network (ANN) can successfully and efficiently handle syndrome and sign information with uncertainty [13]. ANN is a computational model based on the structure and functions of biological neural networks [14]. The remarkable information processing characteristics of the ANN in terms of nonlinearity, fault and noise tolerance, high parallelism, and learning and generalization capabilities contribute to uncertain information processing and quantitative analysis. Furthermore, model-based reasoning (MBR) methods based on machine learning or ANN can successfully process syndrome and sign information with uncertainty to make a precise and accurate diagnosis in integrative medicine.

As mentioned previously, syndrome and sign information or relative information can be extracted from the EMRs, and content texts can be mapped to computability vectors using NLP techniques. Furthermore, MBR methods can be used to create a computer-aided system to support the diagnosis in integrative medicine. However, only a few studies have been conducted on MBR methods with EMRs and NLP to support the diagnosis in integrative medicine. Fortunately, our previous work was carried out to analyze syndrome patterns and syndrome elements in lung diseases based on real-world EMR data [5]. This study aimed to explore MBR algorithms in the diagnosis in integrative medicine based on EMRs and NLP techniques applied on lung disease data sets. We also estimated the associations among the factors of sample size, number of syndrome pattern type, and diagnosis in modern medicine using the MBR algorithms.

## Methods

### Analysis of Workflow

**Figure 1:**
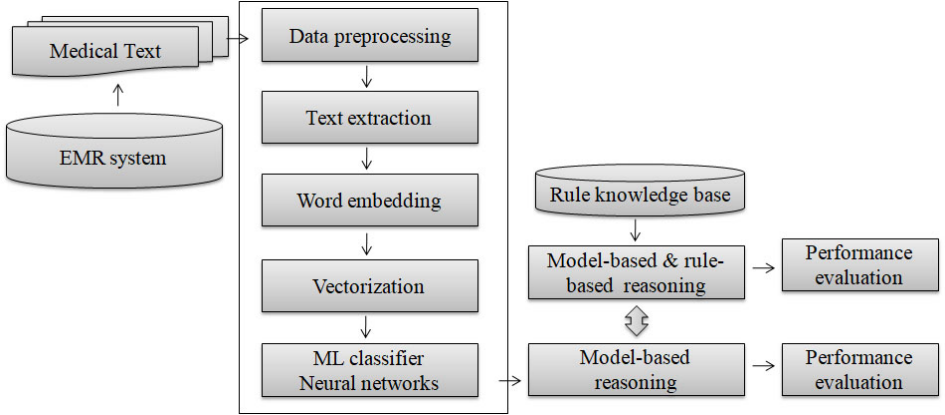
Workflow of the analysis of MBR methods in the diagnosis in integrative medicine based on EMRs and NLP. EMR: electronic medical record; MBR: model-based reasoning; ML: machine learning; NLP: natural language processing.

The workflow of the analysis of the MBR methods in the diagnosis in integrative medicine based on EMRs and NLP is illustrated in Figure 1. The EMRs on lung diseases were exported from the hospital information system, and the syndrome and sign information and relative information were extracted as a text format. The corresponding syndrome pattern diagnosis, clinical diagnosis in modern medicine, and syndrome elements were extracted and saved to the database with the structure data according to the unique code of patients. The content texts of the syndrome and sign information were mapped to the computability vectors through word embedding. The classification models that include the vectors of syndrome and sign information and syndrome patterns or syndrome elements were developed using machine learning or neural network methods. MBR algorithms were developed on the basis of classification models concerning the syndrome pattern, and the model-based and rule reasoning algorithms were developed using the classification models and rule knowledge based on the combination of syndrome elements and syndrome patterns. The performances of the MBR methods in the diagnosis of lung diseases in integrative medicine have been evaluated and compared (for the main program codes for the module, please see [15]).

### Data Collection and Processing

In our previous real-world study on the syndrome pattern and syndrome element of lung disease, EMRs were collected from lung disease wards in 5 hospitals [5]. A data set consisting of 14,075 medical records of clinical cases from 4 hospitals was assigned as the development data set, and it was divided into the train data set and the test data set at a ratio of 4:1. Another independent data set comprising 1000 medical records of clinical cases from a hospital was set as the external test data set. The information comprised patients’ identity number, ward number, admission time, admission notes, first medical records, general medical records, discharge note, diagnosis of syndrome pattern, and diagnosis in modern medicine. In this work, we selected 10 common syndrome pattern types and 8 common lung diseases in the lung disease wards. Nine syndrome element types were generated and combined with the corresponding 10 syndrome pattern types.

### Medical Information Extraction

**Figure 2:**
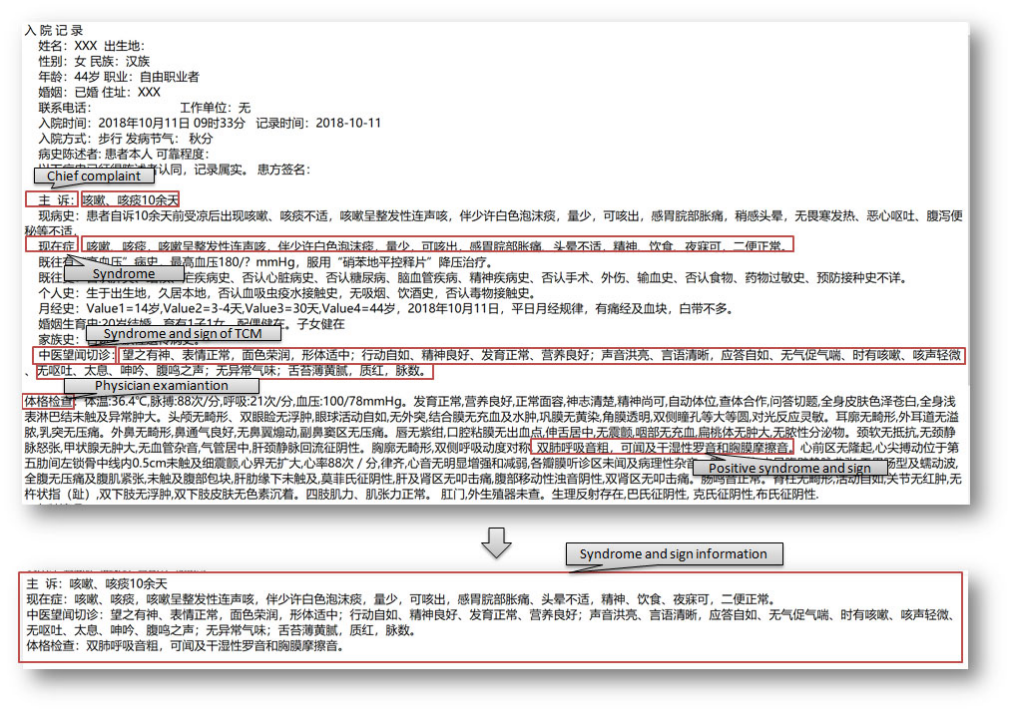
The Chinese text information on the chief complaints, syndromes, and positive signs in the chest, tongue, and pulse that was extracted from the admission notes, first medical records, and discharge records. TCM: traditional Chinese medicine.

The Chinese text information on the chief complaints, syndromes, and positive signs in the chest, tongue, and pulse was extracted from the admission notes, first medical records, and discharge records (Figure 2). The extracted Chinese text information was combined into contexts called “four diagnoses in TCM.” The contexts of the syndromes and signs underwent word-cutting process to split them into tokens. In this work, the first corpus included the context of syndrome and sign information. In the analysis of the diagnosis in modern medicine and syndrome pattern diagnosis, another corpus included an additional token of diagnosis in modern medicine.

### Word2Vec

Word embedding is an NLP feature-learning technique in which words are mapped to vectors of real numbers [16]. Word embedding involves mathematical embedding from a space with 1 dimension per word to a continuous vector space with a much lower number of dimensions. The Word2Vec model is an NLP system that is used to produce word embedding, which takes a large corpus of text as its input and produces a vector space, and each unique word in the corpus is assigned a corresponding vector in the space [16]. The Word2Vec model generates vectors for each word present in a document. In this study, the corpus from a Chinese language Wikipedia dump, which is available at [17], was used to pretrain the word vector model. The parameters utilized with the Word2Vec model were developed for dimension reduction into 256 dimension vectors, 5 context windows, and a minimum sentence word count of 10. The Word2Vec model was implemented using the Gensim Python library [18].

### Doc2Vec

The Doc2Vec model is an extension of Word2Vec that constructs embeddings from entire documents or sentences (instead of individual words) to learn a randomly initialized vector for the document (or sentence) along with the words [19]. The Doc2Vec model modifies the Word2Vec algorithm into an unsupervised learning algorithm that produces continuous representations for large blocks of texts, such as sentences, paragraphs, or entire documents. In this work, Doc2Vec was used to produce vectors for texts. The corpus from a Chinese language Wikipedia dump was again used to pretrain the Doc2Vec model. The parameters utilized with the Doc2Vec model were developed in the dimension reduction into 192 dimension vectors, 5 context windows, and a minimum sentence word count of 10. The Doc2Vec model was also implemented using the Gensim Python library.

### Machine Learning

In this work, the 4 different machine learning classifiers algorithms, namely, random forest (RF), extreme gradient boosting (XGBoost), support vector machines (SVMs), and K-nearest neighbor (KNN), were used to develop MBR [20-22]. The 4 algorithms were the classic machine leaning algorithms, which were the best algorithms suitable for classification tasks.

RF, a classic machine learning classifier, is composed of tree predictors, with each tree depending on the values of a random vector sampled independently and having the same distribution for all trees in the forest [23]. RF aims to reduce the tree correlation issue by choosing only a subsample of the feature space at each split. In this work, RF was used on 1000 trees in the forest, and it was implemented using the scikit-learn Python library.

XGBoost is an optimized distributed gradient-boosting system designed to be highly efficient, flexible, and portable [24]. It implements machine learning algorithms under the gradient boosting framework, which attempts to accurately predict a target variable by combining an ensemble of estimates from a set of simpler, weaker models. XGBoost can also be implemented using the scikit-learn Python library.

SVM is a well-known supervised learning model associated with learning algorithms that analyze data used for classification and regression analysis [25]. SVM was useful in text-based classification tasks and is not prone to errors in high-dimensional data sets. In this work, SVM was used with a linear kernel and implemented using the scikit-learn Python library.

The KNN classifier, one of the most popular machine learning algorithms, is based on the Euclidean distance between a test sample and the specified training samples [26]. It is used for data classification that attempts to determine in which group a data point is included by examining the data points around it. In this study, KNN was implemented using the scikit-learn Python library.

### Artificial Neural Network

ANNs, one of the main tools used in machine learning, are a group of models inspired by biological neural networks used for estimating functions that depend on a large number of inputs [13]. ANN algorithms have 2 different classifiers: multilayer perceptron (MLP) and convolutional neural network (CNN). MLP is a feed-forward ANN model that maps sets of input data onto a set of appropriate outputs [27]. It consists of multiple layers of nodes with a nonlinear activation function in a directed graph, with each layer fully connected to the next one. Back-propagation is used as a supervised learning technique in MLP. In this work, MLP was performed with 6 hidden layers, with the nodes per layer varying from 64 to 1024. It was also implemented using the scikit-learn Python library.

CNN is one of the most popular algorithms for deep learning [28]. It is a category of ANN in which a model learns to perform classification tasks directly from images, text, or sound, and it has been proven effective in the areas of text classification and image recognition. CNN comprises one or more convolutional layers with a subsampling step, followed by one or more fully connected layers as in a standard multilayer neural network [29]. In this work, CNN consisted of an embedding layer, a convolutional layer, a max pooling layer, and 2 fully connected layers, and it was implemented using the Keras Python library.

### MBR

In this study, the development of MBR was based on word embedding and machine learning classifiers for syndrome pattern [30,31]. A total of 11 MBR algorithms were used: Word2Vec RF, Word2Vec XGBoost, Word2Vec SVM, Word2Vec KNN, Word2Vec MLP, Word2Vec CNN, Doc2Vec RF, Doc2Vec XGBoost, Doc2Vec SVM, Doc2Vec KNN, and Doc2Vec MLP. These models with multiclass outputs were consistent with the syndrome pattern types. A comparison of the performance of the 11 MBR algorithms was conducted.

### MBR Combined With Rule-Based Reasoning

MBR was based on word embedding and machine learning classifiers for syndrome elements. Nine MBR algorithms were used: Word2Vec RF, Word2Vec XGBoost, Word2Vec KNN, Word2Vec MLP, Word2Vec CNN, Doc2Vec RF, Doc2Vec XGBoost, Doc2Vec KNN, and Doc2Vec MLP. These models with multilabel outputs were consistent with the syndrome element types. The syndrome patterns were generated by combining the syndrome elements, which follow the rule knowledge base of the syndrome elements, with the syndrome pattern. A comparison of the performance of the 9 MBR combined with rule-based reasoning (RBR) algorithms was performed. The rules of combination of TCM elements for TCM syndrome are presented in Multimedia Appendix 1.

### Evaluation

The performances of the MBR algorithms in syndrome pattern were evaluated in the test data set and the external data set using standard metrics, which included accuracy, precision, recall, and F1 score [32]. Moreover, the performances of the Word2Vec CNN MBR algorithms in each syndrome pattern and each syndrome element were evaluated in the test data set using standard metrics. A fivefold cross validation was conducted 20 times on the train data set for each algorithm to estimate the 95% CI for the performance parameters.

The accuracy comparison analysis of the Word2Vec CNN MBR algorithms in corpus 1 and corpus 2 was conducted in different proportions of the sample size of the development data set. In the accuracy analysis of the data set, each group sample size was set as a proportion of total sample size and the number of syndrome pattern type was selected randomly. The linear regression analyses were conducted to evaluate the associations between each group sample size and the number of syndrome pattern type at accuracies of 0.90% and 0.95% of the methods.

### Ethics Approval and Consent to Participate

The study was approved by the Ethics Committee of the Huashan Hospital and performed in accordance with the Declaration of Helsinki.

### Availability of Data and Material

The data sets generated or analyzed during this study are not publicly available due to private information but are available from the corresponding author on reasonable request. Data sets are from the study whose authors may be contacted at the Center of Bioinformatics and Biostatistics, Institutes of Integrative Medicine, Fudan University. The data concerning external test data set and an example of development data set are available online [15].

## Results

### Development and External Data Sets

**Figure 3:**
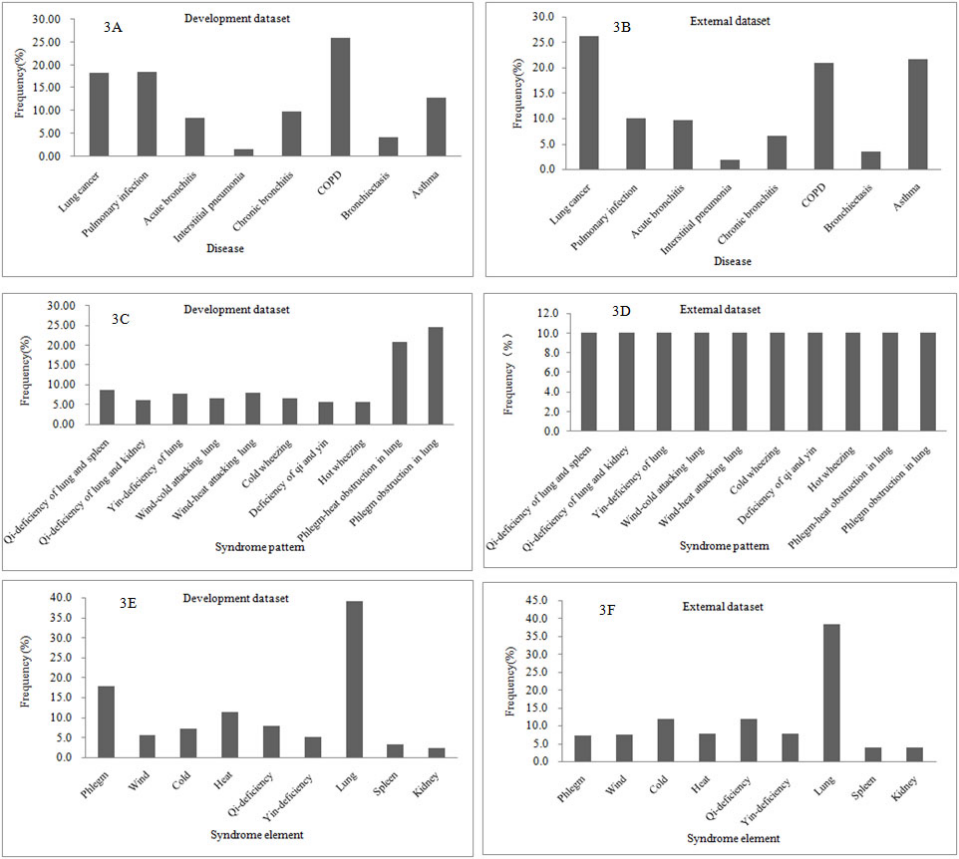
The characteristics of the data set. COPD: chronic obstructive pulmonary disease.

The characteristics of the data set are shown in Figure 3. The development data set consisted of 14,075 medical records of clinical cases, and the external data set had 1000 medical records of clinical cases. Eight common lung diseases were found in the development data set: lung cancer (18.42%), pulmonary infection (18.59%), acute bronchitis (8.39%), interstitial pneumonia (1.66%), chronic bronchitis (9.78%), chronic obstructive pulmonary disease (25.98%), bronchiectasis (4.31%), and asthma (12.88%; Figure 3A). The same common lung diseases with the same proportions were also found in the external data set (Figure 3B). Ten common syndrome pattern types were found in the development data set: qi-deficiency of lung and spleen, qi-deficiency of lung and kidney, yin-deficiency of lung, wind-cold attacking lung, wind-heat attacking lung, cold wheezing, deficiency of qi and yin, hot wheezing, phlegm-heat obstruction in lung, and phlegm obstruction in lung (Figure 3C). The same 10 syndrome pattern types with the same proportions were also found in the external data set (Figure 3D). The development data set had 35,992 syndrome elements for 14,075 syndrome patterns, and a syndrome pattern consisted of 2.56 syndrome elements on average. The development data set included 9 syndrome element types: phlegm, wind, cold, heat, qi-deficiency, yin-deficiency, lung, spleen, and kidney (Figure 3E). A total of 2602 syndrome elements with the same 9 types were found in 1000 syndrome patterns (Figure 3F).

### MBR

In the test data set, the performance analysis of the MBR based on Word2Vec to identify syndrome patterns showed an average accuracy of 0.9397 (95% CI 0.9312-0.9468) in the Word2Vec RF model and 0.9323 (95% CI 0.9213-0.9443) in the Word2Vec ANN model (Table 1). The highest average accuracy was 0.9471 (95% CI 0.9382-0.9549) in the Word2Vec CNN model. The parameters of precision, recall, and F1 score were 0.9478 (95% CI 0.9393-0.9557), 0.9471 (95% CI 0.9382-0.9549), and 0.9470 (95% CI 0.9383-0.9550) in the Word2Vec CNN model, respectively. Similar performance values were found in the corresponding external data set.

**Table 1:** Performance analysis of model-based reasoning methods applied for syndrome pattern diagnosis of lung disease based on Word2Vec in the test and external data sets.

Model and data set		Accuracy, mean (95% CI)	Precision, mean (95% CI)	Recall, mean (95% CI)	F1 score, mean (95% CI)
**Word2Vec + RF^a^**					
	Test	0.9397 (0.9312-0.9468)	0.9411 (0.9331-0.9481)	0.9397 (0.9312-0.9468)	0.9396 (0.9311-0.9468)
	External	0.9121 (0.9001-0.9251)	0.9125 (0.8985-0.9189)	0.9120 (0.9030-0.9220)	0.9118 (0.8988-0.9208)
**Word2Vec + XGBoost^b^**					
	Test	0.8832 (0.8732-0.8942)	0.8844 (0.8714-0.8954)	0.8832 (0.8722-0.8932)	0.8832 (0.8742-0.8972)
	External	0.8720 (0.8641-0.8842)	0.8753 (0.8643-0.8893)	0.8720 (0.8630-0.8860)	0.8728 (0.8598-0.8838)
**Word2Vec + KNN^c^**					
	Test	0.8485 (0.8355-0.8605)	0.8489 (0.8349-0.8569)	0.8485 (0.8355-0.8575)	0.8478 (0.8398-0.8598)
	External	0.8481 (0.8371-0.8611)	0.8514 (0.8404-0.8624)	0.8481 (0.8351-0.8561)	0.8481 (0.8351-0.8591)
**Word2Vec + SVM^d^**					
	Test	0.8172 (0.8062-0.8252)	0.8245 (0.8135-0.8325)	0.8172 (0.8052-0.8312)	0.8161 (0.8071-0.8251)
	External	0.7791 (0.7711-0.7931)	0.8047 (0.7957-0.8177)	0.7791 (0.7681-0.7881)	0.7826 (0.7706-0.7956)
**Word2Vec + MLP^e^**					
	Test	0.9323 (0.9213-0.9443)	0.9326 (0.9226-0.9436)	0.9323 (0.9243-0.9403)	0.9319 (0.9229-0.9409)
	External	0.9203 (0.9101-0.9302)	0.9211 (0.9101-0.9341)	0.9201 (0.9090-0.9340)	0.9193 (0.9063-0.9293)
**Word2Vec + CNN^f^**					
	Test	0.9471 (0.9382-0.9549)	0.9478 (0.9393-0.9557)	0.9471 (0.9382-0.9549)	0.9470 (0.9383-0.9550)
	External	0.9250 (0.9110-0.9360)	0.9277 (0.9153-0.9382)	0.9250 (0.9110-0.9360)	0.9250 (0.9114-0.9362)

^a^RF: random forest.

^b^XGBoost: extreme gradient boosting.

^c^KNN: K nearest neighbor.

^d^SVM: support vector machine.

^e^MLP: multilayer perceptron.

^f^CNN: convolutional neural network.

The performance analysis of the MBR based on Doc2Vec to identify syndrome patterns in the test data set showed the highest average accuracy of 0.8840 (95% CI 0.8730-0.8970) in the Doc2Vec CNN model (Table 2). The parameters of precision, recall, and F1 score were 0.8876 (95% CI 0.8776-0.8976), 0.8840 (95% CI 0.8710-0.8932), and 0.8843 (95% CI 0.8753-0.8973) in the Doc2Vec CNN model, respectively. Similar performance values were found in the corresponding external data set.

**Table 2:** Performance analysis of model-based reasoning methods applied for syndrome pattern diagnosis of lung disease based on Doc2Vec in the test and external data sets.

Model and data set		Accuracy, mean (95% CI)	Precision, mean (95% CI)	Recall, mean (95% CI)	F1 score, mean (95% CI)
**Doc2Vec + RF^a^**					
	Test	0.8320 (0.8198-0.8442)	0.8457 (0.8345-0.8567)	0.8320 (0.8198-0.8442)	0.8337 (0.8217-0.8458)
	External	0.8190 (0.8090-0.8310)	0.8506 (0.8366-0.8610)	0.8190 (0.8110-0.8323)	0.8267 (0.8147-0.8397)
**Doc2Vec + XGBoost^b^**					
	Test	0.7584 (0.7444-0.7724)	0.7682 (0.7602-0.7812)	0.7584 (0.7504-0.7704)	0.7589 (0.7499-0.7719)
	External	0.7270 (0.719-0.7400)	0.7735 (0.7645-0.7835)	0.7270 (0.7130-0.7390)	0.7391 (0.7261-0.7501)
**Doc2Vec + KNN^c^**					
	Test	0.8527 (0.8407-0.8637)	0.8588 (0.8488-0.8668)	0.8527 (0.8407-0.8627)	0.8535 (0.8425-0.8665)
	External	0.8202 (0.8092-0.8282)	0.8246 (0.8116-0.8326)	0.8220 (0.8090-0.8331)	0.8215 (0.8105-0.8295)
**Doc2Vec +SVM^d^**					
	Test	0.6748 (0.6628-0.6848)	0.7424 (0.7334-0.7504)	0.6748 (0.6668-0.6858)	0.7577 (0.7467-0.7667)
	External	0.5820 (0.5700-0.5950)	0.5743 (0.5663-0.5883)	0.5920 (0.5830-0.6033)	0.5288 (0.5168-0.5388)
**Doc2Vec + MLP^e^**					
	Test	0.8840 (0.8730-0.8970)	0.8876 (0.8776-0.8976)	0.8840 (0.8710-0.8932)	0.8843 (0.8753-0.8973)
	External	0.8760 (0.8620-0.8890)	0.8897 (0.8757-0.9027)	0.8760 (0.8630-0.8851)	0.8791 (0.8701-0.8921)

^a^RF: random forest.

^b^XGBoost: extreme gradient boosting.

^c^KNN: K nearest neighbor.

^d^SVM: support vector machine.

^e^MLP: multilayer perceptron.

### MBR Combined With RBR

The performance analysis of the MBR combined with RBR based on Word2Vec in the test data set indicated that the highest average accuracy was 0.9229 (95% CI 0.9099-0.9319) in the Word2Vec CNN model (Table 3). The parameters of precision, recall, and F1 score were 0.9884 (95% CI 0.9744-0.9964), 0.9679 (95% CI 0.9589-0.9809), and 0.9778 (95% CI 0.9698-0.9888) in the Word2Vec CNN model, respectively. Similar performance values were found in the corresponding external data set.

**Table 3:** Performance analysis of model-based reasoning methods in combination with rule-based reasoning methods applied for syndrome pattern diagnosis of lung disease based on Word2Vec in the test and external data sets.

Model and data set			Accuracy, mean (95% CI)	Precision, mean (95% CI)		Recall, mean (95% CI)	F1 score, mean (95% CI)
**Word2Vec + RF^a^**							
	Test	0.9131 (0.8990-0.9261)		0.9934 (0.9814-0.9983)	0.9628 (0.9538-0.9748)		0.9774 (0.9644-0.9864)
	External	0.9040 (0.8903-0.9180)		0.9657 (0.9547-0.9747)	0.9580 (0.9501-0.9721)		0.9617 (0.9477-0.9697)
**Word2Vec + XGBoost^b^**							
	Test	0.7703 (0.7583-0.7803)		0.9666 (0.9556-0.9786)	0.9044 (0.8924-0.9144)		0.9333 (0.9233-0.9433)
	External	0.7980 (0.7871-0.8112)		0.9702 (0.9582-0.9812)	0.9227 (0.9137-0.9337)		0.9444 (0.9364-0.9544)
**Word2Vec + KNN^c^**							
	Test	0.8414 (0.8324-0.8534)		0.9380 (0.9270-0.9502)	0.9254 (0.9164-0.9334)		0.9312 (0.9202-0.9432)
	External	0.8521 (0.8403-0.8612)		0.9441 (0.9321-0.9571)	0.9373 (0.9263-0.9473)		0.9446 (0.9306-0.9556)
**Word2Vec + MLP^d^**							
	Test	0.9052 (0.8930-0.9181)		0.9751 (0.9621-0.9830)	0.9758 (0.9678-0.9858)		0.9752 (0.9652-0.9862)
	External	0.9021 (0.8940-0.9151)		0.9791 (0.9671-0.9911)	0.9780 (0.9660-0.9904)		0.9784 (0.9704-0.9904)
**Word2Vec + CNN^e^**							
	Test	0.9229 (0.9099-0.9319)		0.9884 (0.9744-0.9964)	0.9679 (0.9589-0.9809)		0.9778 (0.9698-0.9888)
	External	0.9160 (0.9030-0.9261)		0.9765 (0.9655-0.9885)	0.9662 (0.9582-0.9782)		0.9698 (0.9608-0.9778)

^a^RF: random forest.

^b^XGBoost: extreme gradient boosting.

^c^KNN: K nearest neighbor.

^d^MLP: multilayer perceptron.

^e^CNN: convolutional neural network.

The performance analysis of the MBR combined with RBR based on Doc2Vec showed that the highest average accuracy was 0.8190 (95% CI 0.8082-0.8281) in the Doc2Vec CNN model (Table 4). The parameters of precision, recall, and F1 score were 0.9550 (95% CI 0.9441-0.9673), 0.9507 (95% CI 0.9387-0.9597), and 0.9524 (95% CI 0.9444-0.9654) in the Doc2Vec CNN model, respectively. Similar performance values were found in the corresponding external data set.

**Table 4:** Performance analysis of model-based reasoning methods in combination with rule-based reasoning methods applied for syndrome pattern diagnosis of lung disease based on Doc2Vec in the test and external data sets.

Model and data set		Accuracy, mean (95% CI)	Precision, mean (95% CI)	Recall, mean (95% CI)	F1 score, mean (95% CI)
**Doc2Vec + RF^a^**					
	Test	0.6410 (0.6281-0.6520)	0.8586 (0.8496-0.8698)	0.9745 (0.9635-0.9865)	0.9049 (0.8939-0.9139)
	External	0.5940 (0.5810-0.6061)	0.9728 (0.9648-0.9828)	0.8002 (0.7892-0.8112)	0.8642 (0.8542-0.8762)
**Doc2Vec + XGBoost^b^**					
	Test	0.6177 (0.6087-0.6307)	0.8525 (0.8415-0.8625)	0.9413 (0.9273-0.9513)	0.8891 (0.8771-0.8981)
	External	0.536 (0.5272-0.5440)	0.9346 (0.9266-0.9486)	0.7863 (0.7763-0.7953)	0.8401 (0.8301-0.8531)
**Doc2Vec + KNN^c^**					
	Test	0.8488 (0.8358-0.8618)	0.9393 (0.9283-0.9523)	0.9503 (0.9383-0.9613)	0.9440 (0.9331-0.9582)
	External	0.8260 (0.8174-0.8383)	0.9203 (0.9073-0.9323)	0.9415 (0.9275-0.9535)	0.9301 (0.9211-0.9401)
**Doc2Vec + MLP^d^**					
	Test	0.8190 (0.8082-0.828)1	0.9550 (0.9441-0.9673)	0.9507 (0.9387-0.9597)	0.9524 (0.9444-0.9654)
	External	0.8031 (0.7911-0.8111)	0.9478 (0.9398-0.9618)	0.9446 (0.9316-0.9546)	0.9444 (0.9314-0.9544)

^a^RF: random forest.

^b^XGBoost: extreme gradient boosting.

^c^KNN: K nearest neighbor.

^d^MLP: multilayer perceptron.

### Word2Vec CNN MBR in Corpus 1 and Corpus 2

**Figure 4:**
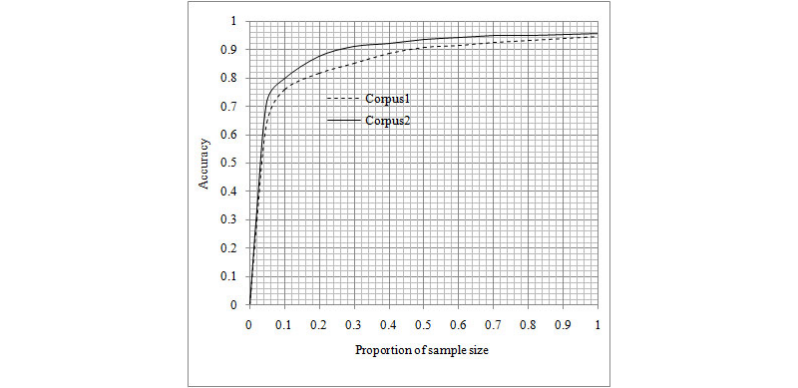
Accuracy and sample size proportions in corpus 1 and corpus 2.

Corpus 1 included the syndrome and sign information without a clinical diagnosis of lung disease, whereas corpus 2 included the syndrome and sign information with a clinical diagnosis of lung disease. A higher average accuracy (0.9584; 95% CI 0.9510-0.9655) was found in the Word2Vec CNN model for syndrome pattern diagnosis in corpus 2 than in corpus 1 (0.9471; 95% CI 0.9382-0.9549) in the test data set (Table 5). Moreover, higher performance parameter values of precision, recall, and F1 score were found in the Word2Vec CNN model for each syndrome pattern diagnosis in corpus 2 than in corpus 1 (Table 5). Similar results were found in the Word2Vec CNN method combined with the RBR model for syndrome pattern diagnosis in corpus 2 in comparison with the model in corpus 1 in the test data set with a full sample size (Table 6). A higher average accuracy of the Word2Vec CNN model was found for syndrome pattern diagnosis in the test data set with different sample sizes in corpus 2 than in corpus 1 (Figure 4).

**Table 5:** Performance analysis of model-based reasoning methods for each syndrome pattern in the test data set with corpus 1 and corpus 2.^a^

Syndrome pattern	Corpus 1				Corpus 2			
	Precision	Recall	F1 score	Support	Precision	Recall	F1 score	Support
Qi-deficiency of lung and spleen	0.9363	0.9514	0.9438	247	0.9957	0.9665	0.9809	239
Qi-deficiency of lung and kidney	0.9362	0.9999	0.9670	176	0.9781	0.9944	0.9861	179
Yin-deficiency of lung	0.9777	0.9733	0.9755	225	0.9902	0.9999	0.9951	203
Wind-cold attacking lung	0.9943	0.9943	0.9956	176	0.9878	0.9999	0.9939	162
Wind-heat attacking lung	0.9899	0.9120	0.9494	216	0.9150	0.9826	0.9476	230
Cold wheezing	0.9724	0.9832	0.9778	179	0.9750	0.9653	0.9701	202
Deficiency of qi and yin	0.9934	0.9804	0.9868	153	0.9932	0.9932	0.9945	147
Hot wheezing	0.9051	0.9931	0.947	144	0.9563	0.9808	0.9684	156
Phlegm-heat obstruction in lung	0.9389	0.9021	0.9201	613	0.9357	0.9125	0.9240	606
Phlegm obstruction in lung	0.9183	0.9344	0.9263	686	0.9461	0.9407	0.9434	691
Average (weighted)	0.9477	0.9471	0.9470	2815	0.9586	0.9584	0.9584	2815

^a^Corpus 1 consists of syndrome and sign information, and corpus 2 consists of syndrome and sign information plus clinical diagnosis information. The average accuracy was 0.9471 (95% CI 0.9382-0.9549) for syndrome pattern in the test data set with corpus 1, and 0.9584 (95% CI 0.9510-0.9655) for syndrome pattern in the test data set with corpus 2.

**Table 6:** Performance analysis of model-based reasoning methods in combination with rule-based reasoning methods for each syndrome element in the test data set with corpus 1 and corpus 2.^a^

Syndrome element	Corpus 1					Corpus 2			
	Precision	Recall	F1 score	Support	Precision		Recall	F1 score	Support
Phlegm	0.9907	0.9538	0.9719	1233	0.9935		0.9951	0.9943	1233
Wind	0.9926	0.9218	0.9559	435	0.9953		0.9770	0.9861	435
Cold	0.9800	0.9722	0.976	503	0.996		1.000	0.998	503
Heat	0.9704	0.8903	0.9286	811	0.9675		0.9174	0.9418	811
Qi-deficiency	0.9616	0.9756	0.9686	616	0.9871		0.9935	0.9903	616
Yin-deficiency	1.000	0.9851	0.9925	403	0.9975		0.9801	0.9887	403
Lung	1.000	1.000	1.000	2815	1.000		1.000	1.000	2815
Spleen	0.9644	0.9457	0.955	258	0.9771		0.9922	0.9846	258
Kidney	0.9882	0.9825	0.9853	171	0.9826		0.9883	0.9854	171
Average (weighted)	0.9885	0.968	0.9779	7245	0.9922		0.9863	0.9892	7245

^a^Corpus 1 consists of syndrome and sign information, and corpus 2 consists of syndrome and sign information plus clinical diagnosis information. The average accuracy was 0.9229 (95% CI 0.9099-0.9319) for syndrome pattern in the test data set with corpus 1, and 0.9559 (95% CI 0.9429-0.9699) for syndrome pattern in the test data set with corpus 2.

### Association of Accuracy and Sample Size With Syndrome Pattern Type

**Figure 5:**
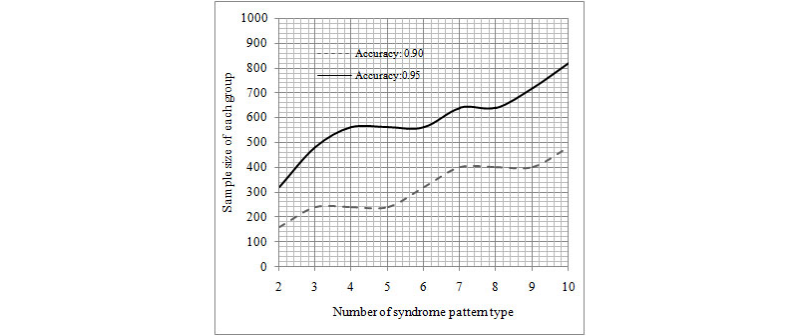
Sample size of each group.

We performed an average accuracy analysis in the development data set classified by the number of syndrome pattern type and each group’s sample size. The results showed that the average accuracy increased with the increase in sample size of each group and decreased with the increase in number of syndrome pattern (Table 7). The linear regression analysis showed that each group’s sample size was significantly associated with the number of syndrome pattern with an accuracy of 0.90 (Y = 34.39 × X + 109.43, *P*<.001, where Y is each group’s sample size and X is the number of syndrome pattern type) and 0.95 (Y = 48.55 × X + 296.78, *P*<.001, where Y is each group’s sample size and X is the number of syndrome pattern type), respectively (Figure 5).

**Table 7:** Average accuracy analysis grouped by sample size of each group and number of syndrome pattern type.^a^

Each group sample size	N=2	N=3	N=4	N=5	N=6	N=7	N=8	N=9	N=10
16	0.5714	0.4001	0.3876	0.3122	0.2521	0.3113	0.3076	0.2068	0.1875
40	0.6575	0.5001	0.4375	0.3511	0.2916	0.3751	0.3751	0.2916	0.2251
64	0.7238	0.6412	0.5384	0.5125	0.4636	0.4444	0.4174	0.4127	0.3921
80	0.8751	0.7291	0.6406	0.6311	0.5521	0.4732	0.5468	0.4513	0.4001
160	*0.9375*	0.8542	0.8437	0.8432	0.8345	0.7901	0.7621	0.7577	0.7325
240	0.9375	*0.9097*	*0.9014*	*0.9011*	0.8993	0.8482	0.8515	0.8487	0.8083
320	*0.9658*	0.9114	0.9074	0.9151	*0.9227*	0.8973	0.8984	0.8836	0.8515
400	0.9688	0.9433	0.9384	0.9281	0.9301	*0.9266*	*0.9023*	*0.9025*	0.8929
480	0.9752	*0.9553*	0.9414	0.9412	0.9418	0.9464	0.9444	0.9234	*0.9135*
560	0.9762	0.9583	*0.9534*	*0.9521*	*0.9532*	0.9482	0.9487	0.9394	0.9304
640	0.9776	0.9653	0.9633	0.9661	0.9626	*0.9526*	*0.9619*	0.9456	0.9354
720	0.9786	0.9708	0.9688	0.9712	0.9709	0.9672	0.9678	*0.9591*	0.9356
800	0.9813	0.9776	0.9756	0.9735	0.9739	0.9785	0.9734	0.9597	0.9429

^a^The first average accuracy was arrived at 0.90 and 0.95 and corresponding values are presented in italics.

## Discussion

### Principal Findings

We developed MBR methods for diagnosis of lung diseases in integrative medicine based on a real-world EMR data set with NLP. In our previous studies, we accumulated large-scale real-world data for artificial intelligence on integrative medicine. In this work, real-world medical records of clinical cases were used to develop models, and medical texts were mapped to vectors of real numbers that a computer could process. CNN approaches can automatically extract features from word vectors, thus contributing to the high performance of MBR methods in syndrome pattern diagnosis for diagnosis of lung diseases in integrative medicine. To the best of our knowledge, this study is the first to investigate MBR methods for diagnosis in integrative medicine on a large real-world data set using NLP and deep learning methods in China. These MBR methods can be recommended for a clinical decision-making system and can also provide a novel approach for diagnosis in integrative medicine. This work would be of significance for applications of artificial intelligence on integrative medicine.

An interesting finding is the high performance of the MBR methods for syndrome pattern diagnosis in integrative medicine. The best Word2Vec CNN MBR method for syndrome pattern diagnosis in integrative medicine had an accuracy of 0.9471 and 0.9250 in the development and external data sets, respectively. Word embedding and CNN contributed to the high performance. Word embedding techniques can map texts to computability vectors, which can perform text analysis with quantitative analysis. CNN can automatically extract features from medical texts, significantly contributing to the performance of the MBR. Additionally, the diagnosis information of modern medicine being added to the corpus enhances the accuracy of the syndrome pattern diagnosis in integrative medicine with reasoning, thus indicating that physicians can more efficiently make a syndrome pattern diagnosis after determining the diagnosis in modern medicine.

We performed an association analysis to evaluate the relationship between the number of syndrome pattern type and each group’s sample size for the accuracy of MBR algorithms. Moreover, we conducted a linear regression analysis to estimate the linear function of each group’s sample size and syndrome pattern type at an accuracy of 0.95. Only a few studies reported on the quantitative associations. In the Word2Vec CNN MBR algorithms at an accuracy of 0.95, the smallest group sample size was 300 for 2 syndrome pattern types, and for each group the sample size was at least 800 for 10 syndrome pattern types. According to the linear model, the Word2Vec CNN MBR method based on each group’s sample size of at least 1200 showed high performance in syndrome pattern with 20 types. A total of 400 common syndrome pattern types were grouped into 20 systems in integrative internal medicine. A total of 25,000 medical records of clinical cases could satisfy the Word2Vec CNN MBR methods in syndrome pattern diagnosis in an integrative system at an accuracy of 0.95. A total of 500,000 medical records of clinical cases could satisfy the Word2Vec CNN MBR methods in the diagnosis of 400 syndrome patterns in the entire integrative internal medicine at an accuracy of 0.95. We could thus combine data-driven artificial intelligence and knowledge-driven artificial intelligence for developing an intelligent clinical decision system on integrative medicine.

Interestingly, the combination of MBR and RBR methods applied for syndrome pattern diagnosis in integrative medicine showed high performance. Specifically, Word2Vec CNN MBR combined with RBR methods had an accuracy of 0.9559 in syndrome pattern diagnosis in corpus 2 with additional information on modern medicine diagnosis. This reasoning method showed a more understandable and clearer knowledge of lung diseases for physicians in comparison with the Word2Vec CNN MBR methods. Moreover, it was more suitable for users of or physicians practicing integrative medicine. Generally, a hybrid reasoning is more suitable for application in clinical practice. The data- and knowledge-driven artificial intelligence contributed to the hybrid reasoning, which has the advantages of high performance reasoning and being explainable for clinicians. In clinical practice, the TCM elements reasoning could be used for TCM diagnosis or differentiation.

Although this study used novel methods to develop MBR in syndrome pattern diagnosis in integrative medicine, it has several limitations. First, we selected only 10 of the 20 common syndrome pattern types in lung diseases, partly because the other 10 syndrome pattern types did not have enough medical records of clinical cases. Therefore, future studies should use comprehensive syndrome patterns in lung diseases or other systems. Second, the size of the corpus for pretrained word vectors was not large to cover all Chinese words or special items on lung diseases.

### Conclusion

MBR methods based on Word2Vec CNN showed high performance in syndrome pattern diagnosis of lung diseases in integrative medicine. The parameters of each group’s sample size, syndrome pattern type, and clinical diagnosis of lung diseases were associated with the performance of the methods. The hybrid reasoning with data- and knowledge-driven artificial intelligence could well contribute to the development of medical artificial intelligence on integrative medicine. We aim to develop a clinical diagnosis or decision-making model with knowledge graph and hybrid reasoning to better combine data- and knowledge-driven artificial intelligence on integrative medicine in the near future.

## Appendix

Multimedia Appendix 1 Rule knowledge base.

## Abbreviations

ANN: artificial neural network
CNN: convolutional neural network
EMRs: electronic medical records
KNN: K-nearest neighbor
MBR: model-based reasoning
MLP: multilayer perceptron
NLP: natural language processing
RBR: rule-based reasoning
RF: random forest
SVM: support vector machine
TCM: traditional Chinese medicine
XGBoost: extreme gradient boosting
